# Microsurgical Treatment and Follow-Up of KOOS Grade IV Vestibular Schwannoma: Therapeutic Concept and Future Perspective

**DOI:** 10.3389/fonc.2020.605137

**Published:** 2020-11-20

**Authors:** Sae-Yeon Won, Andreas Kilian, Daniel Dubinski, Florian Gessler, Nazife Dinc, Monika Lauer, Robert Wolff, Thomas Freiman, Christian Senft, Juergen Konczalla, Marie-Therese Forster, Volker Seifert

**Affiliations:** ^1^ Department of Neurosurgery, University Hospital, Goethe-University, Frankfurt am Main, Germany; ^2^ Department of Neuroradiology, University Hospital, Goethe-University, Frankfurt am Main, Germany; ^3^ Department of Radiosurgery, University Hospital, Goethe-University, Frankfurt am Main, Germany

**Keywords:** vestibular schwannoma, facial nerve functional outcome, hearing nerve, KOOS IV, microsurgical treatment

## Abstract

**Purpose:**

Surgery of KOOS IV vestibular schwannoma remains challenging regarding the balance of extent of tumor resection (EoR) and functional outcome. Our aim was to evaluate the outcome of surgical resection and define a cut-off value for safe resection with low risk for tumor regrowth of KOOS IV vestibular schwannoma.

**Methods:**

All patients presenting at the authors’ institution between 2000 and 2019 with surgically treated KOOS IV vestibular schwannoma were included. Outcome measures included EoR, facial/hearing nerve function, surgical complications and progression of residual tumor during the median follow-up period of 28 months.

**Results:**

In 58 patients, mean tumor volume was 17.1 ± 9.2 cm^3^, and mean EoR of 81.6 ± 16.8% could be achieved. Fifty-one patients were available for the follow-up analysis. Growth of residual tumor was observed in 11 patients (21.6%) followed by adjuvant treatment with stereotactic radiosurgery or repeat surgery in 15 patients (29.4%). Overall serviceable hearing preservation was achieved in 38 patients (74.5%) and good facial outcome at discharge was observed in 66.7% of patients, significantly increasing to 82.4% at follow-up. Independent predictors for residual tumor growth was EoR ≤ 87% (OR11.1) with a higher EoR being associated with a very low number of residual tumor progression amounting to 7.1% at follow-up (p=0.008).

**Conclusions:**

Subtotal tumor resection is a good therapeutic concept in patients with KOOS IV vestibular schwannoma resulting in a high rate of good hearing and facial nerve function and a very low rate of subsequent tumor progression. The goal of surgery should be to achieve more than 87% of tumor resection to keep residual tumor progression low.

## Introduction

Over the past decades, there were notable shifts in the management strategy of vestibular schwannoma. There was a significant decrease of microsurgical treatment, probably due to the increase in the availability of less invasive procedures such as radiosurgery and radiotherapy as well as a significant decrease of the tumor size at the time of diagnosis resulting in a shift towards an observational strategy (1, 2). Nevertheless, grade IV (KOOS classification) vestibular schwannoma, i.e. vestibular schwannoma with a diameter ≥ 3cm and brainstem compression, remains the domain of primary microsurgical or combined radiosurgical-microsurgical treatment presenting unique challenges (3, 4). Whereas the ideal goal is total tumor resection, this often leads to permanent dysfunction of facial or lower cranial nerves (5, 6). Thus, the question arises if it is worthy to perform aggressive surgery or to follow the concept to subtotal resection in order to preserve facial nerve function and leave some residual tumor for radiosurgical treatment.

Several studies investigated the surgical and functional outcome of vestibular schwannoma, but there is paucity of studies focusing solely on KOOS IV vestibular schwannoma (7–9). Recently, a study by Zumofen et al. reported on 44 patients with KOOS IV tumors observing an excellent rate of facial nerve preservation under intentional subtotal resection with a reasonable regrowth rate of remnants (6). As our clinical concept is similar to the study by Zumofen et al., the purpose of this study was to evaluate the validity of current treatment strategies regarding anatomical and functional outcome after microsurgical treatment in a larger cohort of patients with KOOS IV vestibular schwannoma. In addition, we aimed at defining an ideal cut-off value of extent of tumor resection (EoR) intending to result in a low risk of residual tumor progression while preserving facial or cranial caudal nerve function.

## Methods

All patients with KOOS grade IV vestibular schwannoma, who presented at the corresponding author´s institute between 2000 and 2019, were retrospectively enrolled into the study, and their data on clinical and radiological findings were entered into a database. The study was conducted in accordance with the ethical standards laid down in the Declaration of Helsinki after approval of the local ethics committee of Goethe University Frankfurt (approval number 4/09). Written informed consent of each patient was waived for this study.

Beyond baseline demographics, symptoms at admission, data on the operative procedure, EoR, complications, adjuvant treatment and on cranial nerve function at discharge and follow-up were assessed. Two independent clinicians (A.K., M.L.) evaluated pre/postoperative and follow-up magnetic resonance imaging (MRI) scans. The first follow-up MRI was conducted 3 months after surgery, and thereafter MRI was performed at least once per year. Prior to and after surgery, all patients were discussed in our interdisciplinary tumor board to decide upon their individual primary treatment modality and postoperative adjuvant therapy. Tumor volumes were measured assessed by an extracted MRI data set (MRI T1-Gd) with BrainLab^®^ software tool (BrainLab AG, Release date 2013, iPlan^®^ Cranial, Version 3.0, Feldkirchen, Germany) allowing for semiautomated volumetric measurements after outlining tumor borders. Moreover, the presence of perilesional edema was evaluated *via* MRI T2 sequence in 3 categories, as previously described: perifocal, uni- and bilateral (10). Hydrocephalus was defined by Evan´s ratio >0.3; Evan´s ratio was calculated as maximum frontal ventricle width divided by maximum parietal width.

Regarding surgical technique, all operations were performed applying a retrosigmoidal approach and under intraoperative monitoring of motor and sensory evoked potentials. Moreover, intraoperative direct stimulation of the facial nerve and, if applicable, of lower cranial nerves, was performed (11). The internal acoustic meatus was remained unopened in all cases; since our concept was to perform maximal tumor resection by minimizing the risk of facial or cochlear nerve affections. Surgery was stopped either according to surgical anatomy or if cranial nerves were located by direct nerve stimulation with a stimulation intensity of 0.1 mA evoking according compound muscle action potentials (12). Extent of tumor resection was assessed by calculating the difference of pre- and postoperative tumor volumes, expressed as percentage. Near-total, subtotal and partial tumor resection were defined as >90–100%, >80–90% and ≤80% of initial preoperative volume reduction.

Facial nerve function was evaluated using the House & Brackmann (HB) scale. As previously described by Samii et al., excellent outcome was defined as HB1-2, good outcome as HB1-3 and HB4-6 as poor outcome (13). Logopedic assessment of lower cranial nerves functions was performed and all affected patients underwent logopedic treatment during their clinical course. Since hearing function was normal in all patients at the contralateral side of tumor, preservation of hearing was not our primary aim. However, in order to assess patients´ hearing outcome a personalized survey was undertaken dividing the outcome simply in 3 categories: normal hearing, hypacusis compared to the contralateral side and anacusis, with functioning hearing in daily activities being defined as serviceable hearing.

Our primary objective of the study was to evaluate surgical outcome (EoR) followed by the analysis of functional outcome (hearing, trigeminal/facial/caudal cranial nerve function) after surgery and at follow-up with correlation to EoR and identification of predictors for residual tumor progression. Thus, a cut-off value of the EoR was calculated by receiver operating characteristic (ROC) curve analysis.

All calculations and analyses were performed using IBM SPSS Statistics^©^ (version 25, IBM Corp., Armonk, NY, USA). For parametric parameters, mean values were calculated whereas for nonparametric parameters, median values with interquartile range (25–75%) were calculated. The cohorts were stratified by the median creating binary parameters and binary parameters were analyzed using a χ^2^-test. In addition, binary logistic regression was employed for multivariable analysis. For multivariate analysis, parameters identified in univariate analysis were included and independent predictors determined. In order to calculate cut-off value of the EoR, ROC-analysis was performed. A p-value <0.05 was considered to be statistically significant.

## Results

In total, 63 patients with surgically treated KOOS IV vestibular schwannoma were identified within a 20-year period. Five patients were excluded due to insufficient radiological and clinical data leaving 58 patients included in the final analysis.

### Baseline Characteristics and Clinical Presentation at Admission

Patients´ median age was 51 years (range 36–63.5) and sex was equally distributed. The majority of patients were at good admission status with a median Karnofsky performance score of 90 (range 60–100). Median length of hospital stay was 11 days (range 8.3–14).

Most common clinical symptoms at admission were hearing loss in 54 patients (93.4%) followed by vertigo in 26 patients (44.8%), facial dysesthesia in 26 patients (44.8%), ataxia in 24 patients (41.4%), imbalance in 16 patients and headache in 10 patients (17.2%). Only 1 patient (1.7%) was asymptomatic at presentation (**Table 1**).

**Table 1:** Baseline characteristics, symptoms at admission and tumor characteristics.

**Number of patients**	58
**Baseline characteristics**	
Age	
-median (yrs)	51 (36–63.5)
Sex ratio (f/m)	1:1.1
KPS (%)	90 (90–97.5)
Length of stay (days)	11 (8.3–14)
**Symptoms at admission**	
Asymptomatic	1 (1.7%)
Hearing loss -hypacusis -anacusis	46 (79.3%) 8 (13.8%)
Imbalance	16 (27.6%)
Vertigo	26 (44.8%)
Ataxia	24 (41.4%)
Tinnitus	11 (19%)
Nystagmus	8 (13.8%)
Headache	10 (17.2%)
Fascial dysesthesia	26 (44.8%)
Dysarthria	4 (6.9%)
**Tumor characteristics**	
***Side ratio (r/l)***	1:1.3
***Architectural features***	
Solid	54 (93.1%)
Cystic	35 (60.3%)
***Radiological features***	
KOOS IV	58 (100%)
Perilesional edema -perifocal -unilateral -bilateral	28 (48.3%) 15 (25.9%) 10 (17.2%) 3 (5.2%)
Brainstem edema	10 (17.2%)
Hydrocephalus	29 (50%)
CSF Capping	20 (34.5%)

### Radiological Tumor Characteristics

**Figure 1:**
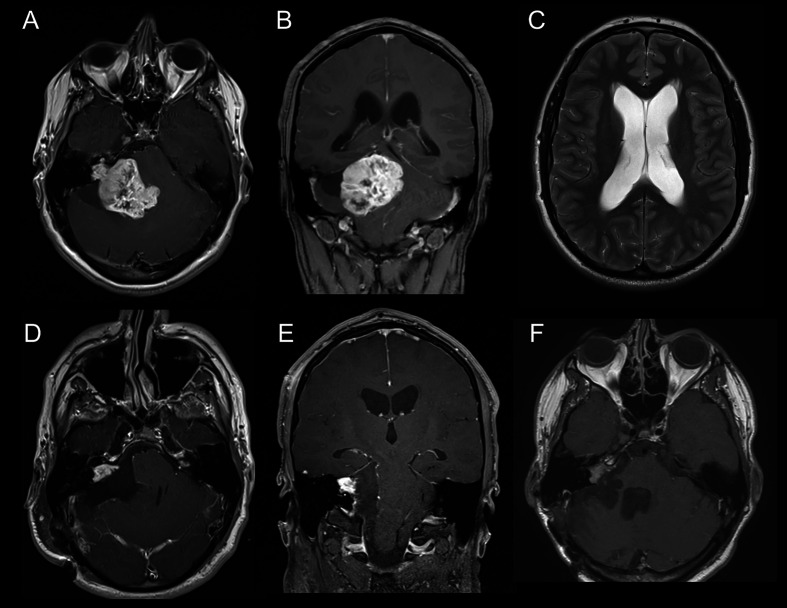
**(A)** Axial slice of MRI-T1Gd showing vestibular schwannoma KOOS IV on the right side. **(B)** Coronar slice of MRI-T1Gd. **(C)** Axial slice of MRI-T2 showing hydrocephalus with Evan´s ratio >0.3. **(D)** Axial slice of postoperative MRI-T1Gd showing residual tumor in the meatus acousticus internus. **(E)** Coronar slice of postoperative MRI-T1Gd. **(F)** Follow-up Image of MRI-T1Gd showing stable residual tumor after adjuvant radiotherapy.

As defined in the inclusion criteria, all 58 patients suffered from KOOS IV vestibular schwannoma with brainstem compression (**Figures 1A, B**) Mean diameter and mean volume of tumors were 36.4 ± 5.6mm (median 35.5mm, range 31.3–39.8) and 17.1 ± 9.2cm^3^ (median 14.1cm^3^, range 11.4–20.9), respectively. The side ratio right to left was 1:1.3. The architecture of tumor was purely solid in 23 patients (39.7%), cystic in 4 patients (6.9%) and combined cystic-solid in 31 patients (53.4%). Preoperative MRI-T2 sequence revealed perilesional edema in 28 patients; among them 15 patients (25.9%) displayed perifocal, 10 patients (17.2%) unilateral and 3 patients (5.2%) bilateral edema. Furthermore, brainstem edema was observed in 10 cases (17.2%). Moreover, in half of the cohort hydrocephalus was diagnosed (**Table 2**) (**Figure 1C**).

**Table 2:** Operative treatment and anatomic outcome at discharge.

Operative treatment	
**Operation positioning**	
Semi-sitting	42 (72.4%)
Park-bench	6 (10.3%)
Side	7 (12.1%)
Prone	3 (5.2%)
**Preoperative prophylactic intervention**	
None	6 (10.3%)
Burr hole	19 (32.8%)
External ventricular drain	33 (56.9%)
**Preoperative volumetric measurement**	
Max. diameter (mean±SD;mm)	36.4±5.6
Volume (mean±SD, cm^3^)	17.1±9.2
**Volumetric measurement of remnant via first postoperative MRI**	
No remnant visible	5 (8.6 %)
0.1<R≤ 1 cm^3^	9 (15.5%)
1 <R≤ 2 cm^3^	13 (22.4%)
2 <R≤ 3 cm^3^	11 (19%)
3 <R≤ 4 cm^3^	7 (12.1%)
4 <R≤ 5 cm^3^	4 (6.9%)
R >5 cm^3^	9 (15.5%)
**Extent of tumor resection (=E)**	
All tumors (mean+SD, n=58)	81.6±16.8%
E>95%	9 (15.5%)
90%<E≤95%	7 (12.1%)
85%<E≤90%	16 (27.6%)
80%<E≤85%	7 (12.1%)
70%<E≤80%	11 (20%)
60%<E≤70%	5 (8.6%)
E<60%	3 (5.1%)
**Major Location of tumor remnant**	
Cisternal	12 (23.5%)
Brainstem	21 (41.2%)
Meatus acousticus internus	18 (35.3%)
**Complications**	
Total number	11 (19%)
CSF leakage with revision/shunt	6 (10.3%)
Sinus thrombosis	2 (3.5%)
Insult	2 (3.5%)
Subdural hematoma	1 (1.7%)

### Operative Procedure and Complications

All patients underwent a retrosigmoidal approach with different patient positioning: the majority of patients, 42 patients (72.4%), were operated in a semi-sitting position and the others either in a park-bench, side or prone position. As per our clinical standard, 33 patients (56.9%) received prophylactic external ventricular drain prior to tumor surgery aiming at intraoperative CSF release, if necessary, and postoperative intracranial pressure monitoring, and 19 patients (32.8%) received a frontal burr hole to facilitate an EVD placement in case of acute hydrocephalus.

In total, 11 patients (19%) had minor or major complications. Corticospinal fluid (CSF) leakage requiring either revision surgery or shunt implantation occurred in 6 patients (10.3%) and cerebellar stroke due to vascular injury or post spatula effect was detected in 2 patients (3.5%) based on radiological finding. Sinus vein thrombosis was diagnosed in 2 patients (3.5%) and infratentorial subdural hematoma in 1 patient (1.7%) (**Table 2**).

### Anatomic Volumetric Outcome at Discharge and Follow-Up

The mean preoperative tumor volume was 17.1 ± 9.2cm^3^ (median 14.1cm^3^ range 11.4–20.9) and a mean EoR of 81.6 ± 16.8% (median 86%, range 78.3–90.1) with a mean residual tumor volume of 3.1 ± 3.1cm^3^ (median 2.3cm^3^, range 1.2–3.8) could be achieved. Complete tumor resection was possible in 5 patients (8.6%), whereas in 53 patients (91.4%) tumor resection had to remain incomplete (**Figures 1D, E**). Residual tumors were located mainly at the brainstem (41.2%) followed by the internal acoustic meatus (35.3%) and along cranial nerves (23.5%). Detailed information is listed in **Table 2**.

Data on follow-up examinations were available in 51 patients (87.9%) assessed after a median time of 28 months (range 4.3–53.8) and a mean time of 33.7 months (SD 35.6). After complete tumor resection, no tumor regrowth was observed during the follow-up. In those 46 patients with residual tumors, stable disease was documented in 21 patients (45.7%), tumor regression in 12 patients (26.1%) and residual tumor progression in 11 patients (23.9%) (**Table 3A**) (**Figure 1F**). In 2 patients, volumetric measurement was not feasible due to missing preoperative radiological data. Analyzing the pre/postoperative volume with residual tumor behavior, a clear correlation was found: Larger preoperative volume and larger postoperative residual tumors were associated with remnant progression, with a mean tumor volume of 6.4 ± 4.0cm^3^ at follow-up. Vice versa, smaller preoperative volume and smaller postoperative residual tumors showed stable disease at follow-up, having a mean tumor volume of 2.6 ± 2.1 cm^3^. Postoperative treatment was initiated in 16 patients (31.4%) according to the recommendation of the local interdisciplinary tumor board: Thus, stereotactic radiosurgery was performed in 11 patients (21.6%), repeated surgery in 4 patients (7.8%) and a combined radiosurgery and operation in 1 patient (2%). Moreover, placement of a ventriculo-peritoneal shunt was necessary due to postoperative cerebrospinal fluid fistula in 6 patients (10.3%) (**Table 3B**).

**Table 3A:** Anatomic outcome at follow-up.

**Follow-up (n=51)** -median (range, days, months)	856 (150.3–1,647.3) / 28 (4.3–53.8)			
	**No R**	**R constant**	**R regression**	**R progression**
**No Remnant (n=5)**	5 (100%)	0 (0%)	0 (0%)	0 (0%)
**Remnant (n=46)**	2 (4.3%)	21 (45.7%)	12 (26.1%)	11 (23.9%)

**Table 3B:** Volumetric analysis of residual tumor at follow-up and postoperative treatment.

**Residual tumor volumetric measurement and extent of resection at follow-up**				
All tumors (mean ± SD, n=49)*	Preoperative	Postoperative	Extent of resection	Last follow-up
No Remnant (n=5)	10.8 ± 1.9 cm3	0 cm3	100%	0 cm3
Remnant constant (n=21)	17.5 ± 10.8 cm3	2.8 ± 1.9 cm3	82 ± 11.2%	2.6 ± 2.1 cm3
Remnant regression (n=12)	16.6 ± 9.4 cm3	4 ± 4.2 cm3	84.9 ± 12.8%	2.8 ± 2.8 cm3
Remnant progression (n=11)	21.3 ± .7.8 cm3	4.3 ± 2.9 cm3	80.1 ± 12.7%	6.4 ± 4.0 cm3
**Postoperative treatment**				
Stereotactic radiosurgery	11 (21.6%)			
Repeat surgery	4 (7.8%)			
Combined	1 (2%)			
Postoperative shunt	6 (11.8%)			

*2 Patients with documented progression of a tumor remnant were lost to follow-up after the first follow-up.

### Predictors for Residual Tumor Progression

In the univariate analysis, residual tumor volume >3cm^3^ and EoR <87% were significant predictors for remnant progression. Patient age <39 years showed a trend towards significance (p=0.06) (**Table 4**). In the multivariate analysis, the only independent predictor for residual tumor progression was EoR <87% (p=0.03; OR 11.1 Cl 95% 1.2–100).

**Table 4:** Predictors for remnant progression.

	Tumor progression	Tumor constant regression	Univariate analysis p-value	Multivariate analysis	
				p-value	OR (Cl 95%)
Number	11	40			
Sex (f)	7 (63.6%)	18 (45%)	0.27		
Age<39	6 (54.5%)	10 (25%)	0.06		
Side (left)	4 (36.4%)	18 (45%)	0.15		
Solid	11 (100%)	37 (92.5%)	0.35		
Cystic	4 (36.4%)	26 (65%)	0.09		
Volume >14cm3	7 (63.6%)	18 (45%)	0.27		
Perilesional edema	4 (36.4%)	18 (45%)	0.61		
-Perifocal	3 (27.3%)	8 (20%)	0.60		
-Unilateral	1 (9%)	7 (17.5%)	0.50		
-Bilateral	0 (0%)	3 (7.5%)	0.35		
-Brainstem	1 (9%)	8 (20%)	0.14		
Hydrocephalus	6 (54.5%)	20 (50%)	0.79		
CSF Capping	4 (36.4%)	12 (30%)	0.69		
Remnant volume (>3cm3)	7 (63.6%)	11 (27.5%)	0.03	0.56	1.6 (0.3-8.9)
Extent of resection < 87%	10 (91%)	20 (50%)	0.02	0.03	11.1 (1.2–100)

### Outcome at Discharge and Follow-Up

Fifty-one patients were eligible for follow-up analysis.

### Hearing Outcome

**Figure 2:**
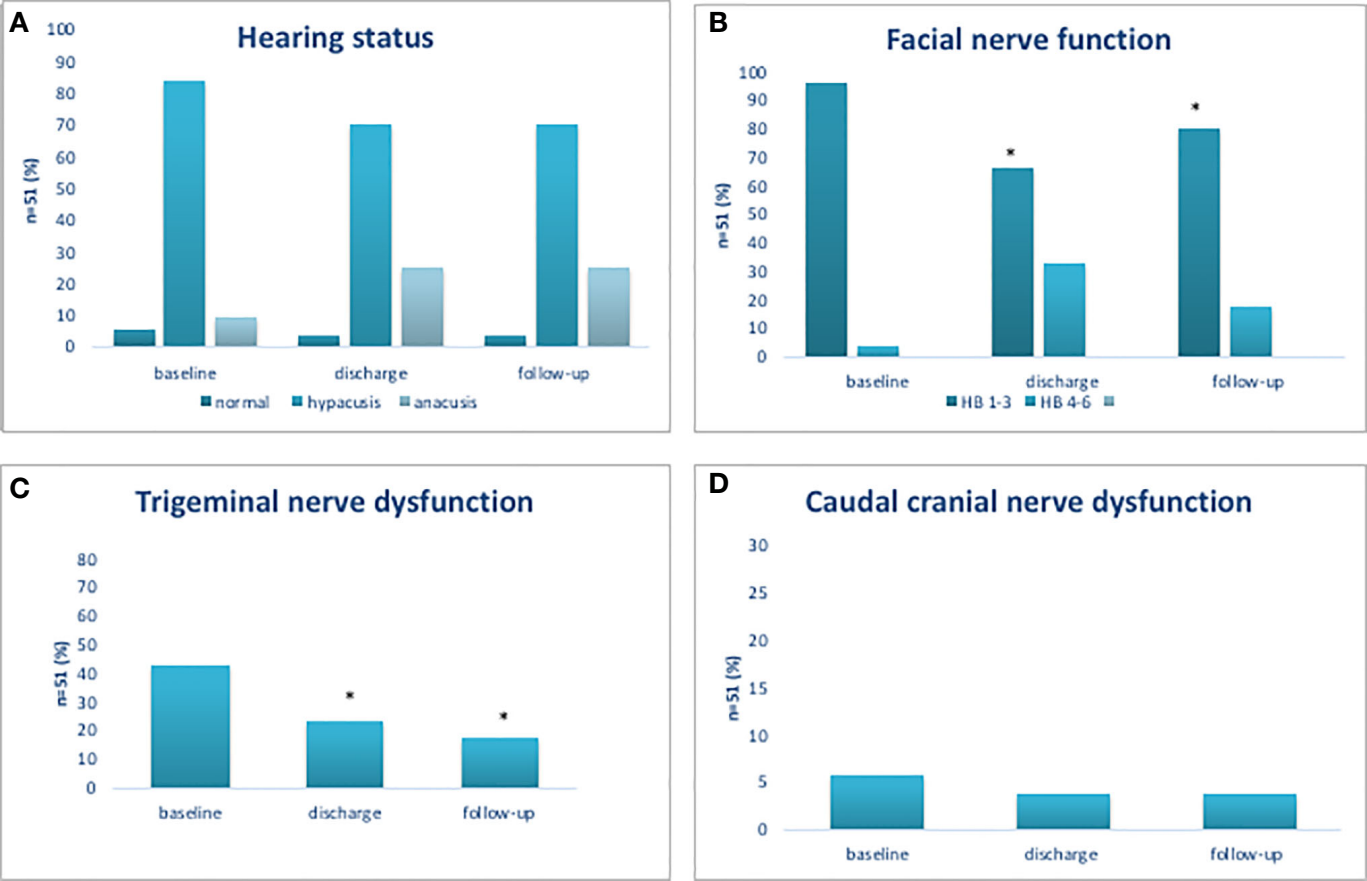
Functional outcome of cochlear **(A)**, facial **(B)**, trigeminal **(C)**, and cranial caudal nerve **(D)**. Functional outcome of cochlear, facial, trigeminal, and cranial caudal nerve. *p < 0.05.

At admission, all patients (100%) had normal hearing status contralateral to the tumor. Ipsilateral hypacusis was present in 43 patients (84.3%), anacusis in 5 patients (9.8%) and 3 patients (5.8%) had normal hearing status. Postoperatively, additional 8 patients suffered from a significant hearing loss (25.5%, p=0.04), not experiencing any improvement or worsening during the follow-up period (**Figure 2A**).

### Facial Nerve Outcome

At admission, 49 patients (96.1%) had good facial nerve function (HB 1–3) and 2 patients (3.9%) had poor facial nerve function (HB 4–6). After surgery, good facial nerve function was observed in 34 patients (96.1 vs 66.7%, p<0.001). At follow-up, the number of patients with good facial nerve function had increased, now present in 42 patients (96.1 vs 82.4%, p=0.01); however, pre-existing facial nerve palsy (HB 5 or 6) had neither improved after surgery nor at follow-up examination (**Figure 2B**).

### Trigeminal and Caudal Crania Nerve Outcome

Among 51 patients, 22 patients (43.1%) displayed trigeminal nerve affection prior to surgery. After surgery, the number of patients with trigeminal dysfunction was significantly reduced (43.1 vs 23.5%, p=0.04) and at follow-up, a further reduction was noted (43.1 vs 17.6%, p=0.005) (**Figure 2C**). Caudal nerve affection was present in 3 patients (5.9%) prior to surgery, in 2 patients (3.9%) thereafter and in 2 patients (3.9%) at follow-up (**Figure 2D**).

### Outcome Depending on Extent of Resection

Favorable outcome of the function of all cranial nerves correlated well with EoR: with increasing EoR the outcome became worse. EoR>90% was associated with good facial nerve function in 10 of 16 patients (62.5%), whereas the best outcome, 10 of 11 patients (90.9%), was achieved in patients experiencing an EoR between 70 and 80%. Regarding trigeminal nerve or cranial caudal nerve function, no severe dysfunctions were observed after surgery. As mentioned above, EoR was an independent predictor for tumor progression, with an EoR of 87% found to be of predictive cut-off value for tumor progression in the ROC-analysis. Analyzing those cohorts with EoR≥87%, good facial nerve function was present in 20 of 28 patients (71.4%), normal trigeminal nerve function in 20 of 28 patients (71.4%) and normal cranial caudal nerve function in 25 of 28 patients (89.3%) at follow-up (**Supplementary Figure 1**). Furthermore, local tumor control was significantly better in patients with EoR≥87% (2 of 28 patients, 7.1%) compared to those with lower EoR (9 of 23 patients, 39.1%) (p=0.008).

## Discussion

Several studies reported gross total resection of giant vestibular schwannoma as the optimal treatment option for tumor control with reasonable rate of favorable outcome (5, 14, 15). In these cases, the risk for long-term recurrence rate was less than 1% within 5 years of follow-up whereas subtotal tumor debulking was reported to have higher recurrence rate at long-term follow-up up to 27.6% (6, 16). On the other hand, the outcome of facial nerve function correlates inversely with EoR, and a high EoR was even identified as an independent predictor for poor facial nerve function (5, 16). Thus, in the era of radiosurgery and radiotherapy, the goal of surgery might be “reasonable” tumor resection by preserving facial nerve function. Accordingly, in the present cohort of patients’ extensive tumor resection was not our aim achieving subtotal resection in the majority of our patients with a mean of 82% tumor volume reduction. In 22% of these patients, tumor progression was observed within 3 years correlating well with other studies (6, 17, 18). Most probably, the rate of volumetric progression could be reduced by adjuvant radiotherapy, however, and according to the recommendation of our local interdisciplinary tumor board, direct postoperative adjuvant radiotherapy was only performed in a small number of patients (6). Patients with small remnants are being rather observed, and radiotherapy is withheld as an alternative treatment for eventual later tumor regrowth.

Several authors advocate increasing recurrence rate depending on follow-up duration: Chen et al. and Carlson et al. reported on a 18–22% recurrence rate after a mean follow-up of 3–4 years, whereas Bloch et al. observed tumor recurrence in 32% of their patients and Fukuda et al. in 55% of their patients after mean follow-up of 4.3 years and 8.7 years, respectively (5, 17, 19, 20). Depending on the volume of residual tumor, the dynamic of recurrence varies, making life time follow-up evaluation often necessary (21). Equally, patients at high risk for tumor recurrence need to be identified, to be offered either adjuvant treatment or an intensive follow-up concept. Therefore, we tried to identify those patients by applying independent predictors for tumor progression.

The primary strengths of the present study are the identification of an EoR ≥ 87% being associated with significantly lower rates of tumor recurrence, and so impacting patient´s long-term outcome. This is in line with other studies favoring near-total removal as the primary goal of KOOS IV vestibular schwannoma surgery (22, 23). Consequently, in patients with less than 87% of volume reduction, early adjuvant treatment should be considered postoperatively.

The second strength of our study is patient´s good clinical outcome. Despite the large size of tumors and their high EoR good facial function was observed in ≥ 80% of patients. Similar results were presented by Zhang et al. reporting on good facial nerve function in 56% of patients after gross total resection and in 79.6–83.3% of patients after near-total to subtotal resection (24). Moreover, Huang et al. reported on a large series of unilateral giant vestibular schwannoma with 75.6% of 657 patients presenting good facial nerve function after gross-total to near-total resection (25). On the other hand, a recent study by Zumofen et al. analyzing particularly KOOS IV vestibular schwannoma even reported on excellent facial nerve outcome (HB I–II) in up to 89% after intended near-total resection (6). Nevertheless, it is not surprising, because the mean tumor volume of the study by Zumofen et al. was 10cm^3^ whereas in our study the mean tumor volume was 1.7 fold higher. It is well known that the outcome of cranial nerve function correlates well with preoperative tumor volume as well as with EoR (26). Macielak et al. have shown that mean preoperative tumor volume ≥ 15cm^3^ results in postoperative excellent postoperative facial nerve function (HB I–II) in 30% of patients whereas a tumor volume < 15cm^3^ comes along with excellent facial function in more than 50% of patients (24, 26). In this context, patients of the present study experienced both very good facial nerve outcome (excellent facial nerve function with HB I–II in 60.1% of patients) and good extent of tumor resection. Moreover, most patients suffered from diminished, but functional hearing prior to surgery, and serviceable hearing could be preserved in over 70% of patients. In the study by Zumofen et.al, the rate of serviceable hearing was 14% after intended near-total resection of KOOS IV vestibular schwannoma, taking into consideration that only 38% of patient had serviceable hearing before surgery (6). The most relevant factor for hearing preservation is the tumor size. Glasscock et al. showed that patients with tumors larger than 2cm had low chance for hearing preservation (13) In particular, several publications reported on low hearing preservation in KOOS IV vestibular schwannoma coming up to 11–15% (6, 25, 27).

Concerning the surgical approach, there are different opinions. In the present series, all patients underwent a retro sigmoidal approach, since a translabyrinthine approach always comes along with a hearing loss of the ipsilateral side. Furthermore, our surgical strategy was to perform subtotal resection without opening the internal acoustic meatus, which might be necessary for good hearing preservation.

To summarize, we think that intended subtotal resection of KOOS IV vestibular schwannoma is a good therapeutic concept to preserve reasonable hearing and good facial nerve function with a reasonable low risk of recurrence in the days of optional adjuvant radiosurgery and radiotherapy following surgery.

In our study, we had a midterm follow-up period, however the course of residual tumor over a longer follow-up period needs to be evaluated as well as long-term quality of life. There were not objective hearing tests performed in our cohort of patients which lead to some difficulty regarding uniform interpretation of hearing outcome. Since the primary goal of the surgery was not to preserve ipsilateral hearing, regular objective hearing tests were waived in our clinical practice. Ultimately, this is a retrospective study inheriting limitations resulting from the retrospective nature.

## Conclusion

The therapeutic concept of subtotal resection of KOOS IV vestibular schwannoma has proven advantageous. Good facial nerve function was achieved in over 80% of patients and a serviceable hearing preservation in 74.6% of patients. The rate of remnant growth was low in patients who benefited of at least 87% of tumor resection amounting to 7%, whereas residual tumor progression was seen in 39% of patients after less than 87% EoR indicating the necessity of adjuvant radiotherapy.

## Data Availability Statement

The original contributions presented in the study are included in the article/**Supplementary Material**. Further inquiries can be directed to the corresponding author.

## Ethics Statement

The studies involving human participants were reviewed and approved by local ethics committee of Goethe University Frankfurt (approval number 4/09). Written informed consent for participation was not required for this study in accordance with the national legislation and the institutional requirements.

## Author Contributions

S-YW: study design, study conception, data extraction, data analysis, statistical analysis, manuscript writing. AK: data extraction, data analysis. DD: study conception, critical review of the manuscript. FG: study design, data analysis and interpretation, statistical analysis, study supervision. ND: data extraction, data analysis, critical review of the manuscript. ML: radiological measurement, critical review of the manuscript. RW: study design, study conception. TF: critical review of the manuscript, study supervision. CS: critical review of the manuscript, study supervision. JK: study conception, critical review of the manuscript, study supervision. M-TF: study design, study conception, interpretation, critical review of the manuscript, study supervision. VS: study design, study conception, critical review of the manuscript, study supervision. All authors contributed to the article and approved the submitted version.

## Conflict of Interest

The authors declare that the research was conducted in the absence of any commercial or financial relationships that could be construed as a potential conflict of interest.

## Supplementary Material

Supplementary Figure 1 Functional outcome of facial, trigeminal and cranial caudal nerve depending on the extent of tumor resection.
