# Supplementary figures and images for: Microsurgical Treatment and Follow-Up of KOOS Grade IV Vestibular Schwannoma: Therapeutic Concept and Future Perspective
Source: Front Oncol. 2020 Nov 20;10:605137. doi: 10.3389/fonc.2020.605137 (PMC7714957; doi:10.3389/fonc.2020.605137)

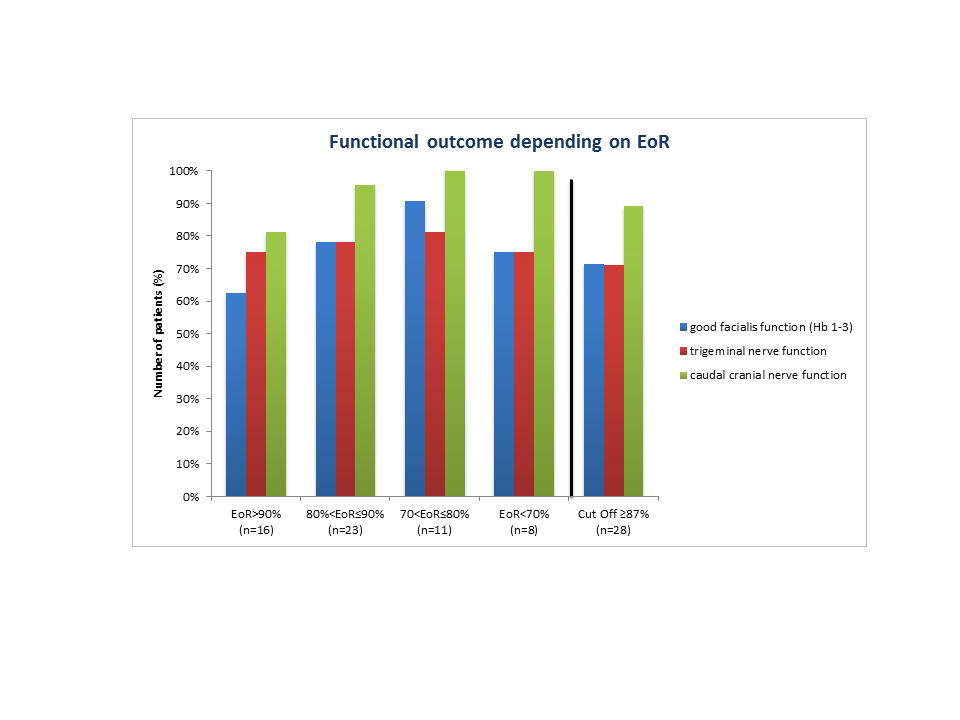

Supplement: Supplementary Figure 1 — Functional outcome of facial, trigeminal and cranial caudal nerve depending on the extent of tumor resection. [file Image_1.tif]
